# Exploring seasonal diurnal surface temperature variation in cities based on ECOSTRESS data: A local climate zone perspective

**DOI:** 10.3389/fpubh.2022.1001344

**Published:** 2022-09-06

**Authors:** Zhipeng Shi, Jun Yang, Ling-en Wang, Fang Lv, Guiyang Wang, Xiangming Xiao, Jianhong Xia

**Affiliations:** ^1^Human Settlements Research Center, Liaoning Normal University, Dalian, China; ^2^School of Humanities and Law, Northeastern University, Shenyang, China; ^3^Jangho Architecture College, Northeastern University, Shenyang, China; ^4^Institute of Geographic Sciences and Natural Resources Research, Chinese Academy of Sciences, Beijing, China; ^5^Urban planning, mapping, and geographical information service center of Dalian, Dalian, China; ^6^Department of Microbiology and Plant Biology, Center for Earth Observation and Modeling, University of Oklahoma, Norman, OK, United States; ^7^School of Earth and Planetary Sciences (EPS), Curtin University, Perth, WA, Australia

**Keywords:** Local Climate Zone, diurnal variation, land surface temperature, ECOSTRESS data, urban morphology, Beijing

## Abstract

High urban temperatures affect city livability and may be harmful for inhabitants. Analyzing spatial and temporal differences in surface temperature and the thermal impact of urban morphological heterogeneity can promote strategies to improve the insulation of the urban thermal environment. Therefore, we analyzed the diurnal variation of land surface temperature (LST) and seasonal differences in the Fifth Ring Road area of Beijing from the perspective of the Local Climate Zone (LCZ) using latest ECOSTRESS data. We used ECOSTRESS LST data with a resolution of 70 m to accurately interpret the effects of urban morphology on the local climate. The study area was dominated by the LCZ9 type (sparse low-rise buildings) and natural LCZ types, such as LCZA/B (woodland), LCZD (grassland), and LCZG (water body), mainly including park landscapes. There were significant differences in LST observed in different seasons as well as day and night. During daytime, LST was ranked as follows: summer > spring > autumn > winter. During night-time, it was ranked as follows: summer > autumn > spring > winter. All data indicated that the highest and lowest LST was observed in summer and winter, respectively. LST was consistent with LCZ in terms of spatial distribution. Overall, the LST of each LCZ during daytime was higher than that of night-time during different seasons (except winter), and the average LST of each LCZ during the diurnal period in summer was higher than that of other seasons. The LST of each LCZ during daytime in winter was lower than that of the corresponding night-time, which indicates that it is colder in the daytime during winter. The results presented herein can facilitate improved analysis of spatial and temporal differences in surface temperature in urban areas, leading to the development of strategies aimed at improving livability and public health in cities.

## Introduction

With the rapid development of urbanization, the proportion of impervious surface in cities is increasing, and these materials usually have higher heat storage capacity and lower reflectance. In addition, the increase in population of cities will also bring about an increase in energy consumption and greenhouse gas emissions. As a result, the temperature of the inner city is higher than that of the surrounding areas, which is called the urban heat island (UHI) effect (1, 2). The UHI affects air quality (3), vegetation phenology (4), and energy and water demand (5–7), while also increasing heat-related human casualties (8). By 2050, 68% of the global population is expected to live in urban areas, according to UN statistics (9). Previous studies have shown that the change of thermal environment will not only affect people's physiological condition, but also have serious psychological effects (10). Meanwhile, global heat wave events are increasing in frequency (11–13), and climate warming have also changed the urban thermal environment to some extent. Therefore, the spatial and temporal patterns and variability of the urban thermal environment must be explored to improve livability and public health in cities for global urban development (14–16).

Based on the location of the heat island, UHI effect can be divided into Subsurface UHI, Surface UHI, Canopy layer UHI and Boundary layer UHI (17, 18). The UHI effect is usually quantified through urban heat island intensity (UHII) (19). It is defined as the difference between urban surface temperature and suburban surface temperature (20). The temperature data are mainly obtained through the temperature data measured by weather stations or the inversion based on satellite remote sensing data. Meteorological station data have high temporal resolution and continuity; however, due to the limitations of observation instruments as well as the number and spatial location of meteorological stations, the data cannot reflect the overall spatial variation of urban temperature comprehensively. For example, Zhang et al. (19) obtained long-time series temperature data from 50 stations for 3 years (2014, 2015, and 2017) to analyze the UHI characteristics of Xi'an during summer daytime; however, on-site measurement methods are often labor-intensive. In contrast, remote sensing inversion can be used to obtain large-scale surface temperature data in a short period of time and analyze the characteristics of the urban thermal environment on different scales (21–23). Depending on the satellite orbit, remote sensing data can be divided into near-polar orbit satellite remote sensing data and geostationary satellite remote sensing data (24, 25). The commonly used MODIS, ASTER, and Landsat data are all near-polar orbit satellite data, which are acquired in essentially the same amount of time for a given location. Therefore, near-polar orbit satellite data can perform long-term comparisons of UHI effects.

The difference of surface temperature between cities and adjacent non-urban areas is called surface urban heat island (SUHI), which is usually calculated by land surface temperature (LST) measured by satellite (26). Different scholars have studied the changes of surface temperature from many perspectives, including seasonal and diurnal surface temperature variation in cities. From the perspective of spatial scale, these studies are mainly divided into global scale, regional scale and individual city surface temperature variation (27–29), and from the time perspective, mainly into diurnal, seasonal and inter-annual variation (30–32). Most scholars use MODIS data for research to ensure good consistency in space and time series (33, 34). Some scholars combine Landsat data with MODIS data, for instance, Md. Omar Sarif and Rajan Dev Gupta used multi-time Landsat and MODIS Terra to study the ecological status of Prayagraj city and its surrounding environment during summer and winter (35). However, MODIS data have certain deficiencies when used in the research of some small-scale areas. First, in terms of spatial resolution, MODIS data are suitable for large-scale studies of surface temperature variability (36, 37), while ASTER and Landsat satellite inversions are more suitable for urban-level studies of thermal environments (38). In terms of diurnal surface temperature data acquisition, MODIS and ASTER data have low spatial resolution and cannot accurately reflect intra-urban variability, despite the availability of night-time data (39–41) and good applications for observing UHI effects in large cities or metropolitan areas (42). In addition to polar orbiting satellites, geostationary satellites can also acquire diurnal surface temperature data and have shown good accuracy (43). For satellites such as the Geostationary Operational Environmental Satellite (GOES) (44), Meteosat (45), and Meteosat Second Generation (MSG) (46), it is often difficult to obtain an accurate picture of the thermal environment inside cities due to spatial resolution limitations. For example, Chang et al. (20) explored the diurnal UHII cycle in Boston based on GOES-R surface temperature data, but its spatial resolution was only 2 km.

In 2018, NASA developed the ECOsystem Spaceborne Thermal Radiometer Experiment on Space Station (ECOSTRESS), which is mainly used to study the response patterns of plant temperature to water. With a short revisit period of 3–5 days, a resolution of 70 m, and the ability to acquire LST images at different times during the day and night, ECOSTRESS data have a high spatial and temporal resolution, and ECOSTRESS LST and emissivity (LST&E) data have been widely applied to research urban thermal vulnerability (47), agricultural monitoring (48), and surface temperatures in volcanic regions (49) with good accuracy. Thus, retrieving ECOSTRESS data for surface temperature can provide a more comprehensive understanding of the spatial and temporal impacts of urban morphology on LST. Understanding the influence of urban landscape patterns on LST heterogeneity is essential for improving the urban thermal environment (50, 51); however, the urban/rural division traditionally only distinguishes between urban and suburban areas, limiting research at the local urban scale. To overcome these limitations, Stewart and Oke (52) proposed the Local Climate Zone (LCZ) scheme, which divides the landscape into 10 “built types” and 7 “natural types,” each of which characterizes land cover, surface properties, and human activity. Li et al. (53) based on the classification of LCZ, analyzed the variation rules of surface temperature (LST) and frontal area index (FAI), and put forward the suggestion of urban cooling by wind. In addition, Xie et al. (54) produced the LCZ map of the Guangdong–Hong Kong–Macao Greater Bay Area for a long time series to analyze the spatial and temporal changes of urbanization gradient and urban form from a three-dimensional perspective. Therefore, the LCZ scheme represents the thermal environment of homogeneous surfaces within cities in detail and has been widely applied to the study of urban thermal environments (36, 55–57) and urban wind environments (58, 59).

In summary, due to the high heterogeneity within cities and availability of satellite data, the current research on LCZ–LST is mostly based on near-polar orbit satellite data, and the use of geostationary satellite remote sensing data is relatively rare. However, the ECOSTRESS experiment can capture temperature data at high resolution during the day and night; thus, it can better observe the heat phenomenon inside the city better. Therefore, this study mainly includes three objectives: (1) use 70 m resolution ECOSTRESS data to obtain night and day surface temperatures for all seasons and explore the spatial variability between surface temperatures; (2) classify different LCZ types using Geographic Information System (GIS) identification based on remote sensing and building data; and (3) spatially overlay LCZ and LST to analyze the spatial variability of different LCZs between various seasons and between day and night.

## Data and methods

### Study area

**Figure 1:**
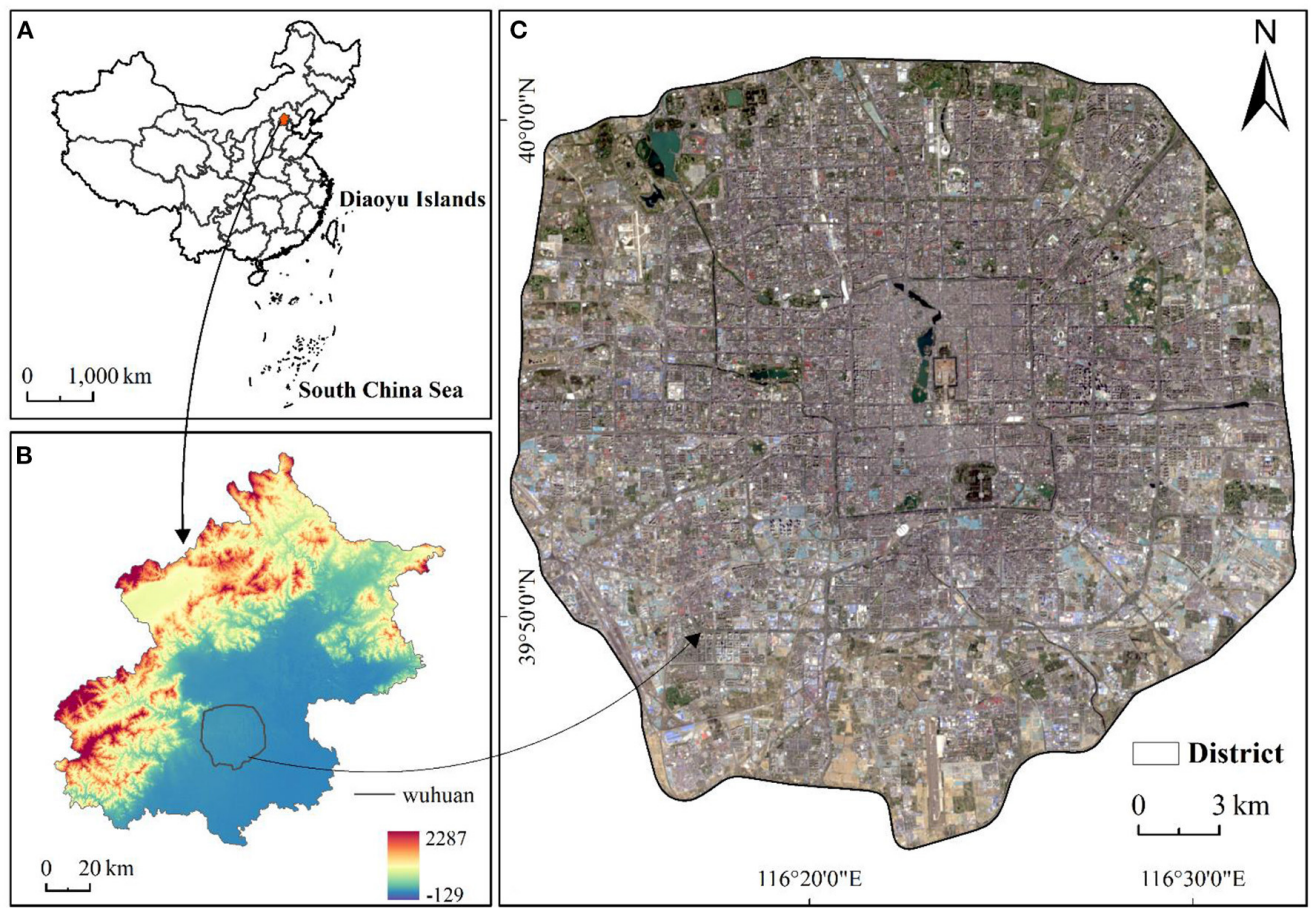
The location of the study area. **(A)** The red shaded area represents the location of the study area in China, **(B)** Beijing decorated with elevation data, and **(C)** satellite images of the study area.

Beijing is located at 115.7–117.4°E, 39.4–41.6°N. The total area is ~16,410.54 km^2^, This study takes the Fifth Ring Road as the research area. The main reason for this selection is that according to the 7th National Population Census (2021), the permanent population of Beijing is about 21.89 million. The permanent population in the central urban area, which accounts for only 4% of the total area, is about 10.99 million and accounts for 50.2% of the total population. The limited land area and the increasing population exert great pressure on the urban thermal environment, especially during summer season. In addition, the area has little relief and shows minor changes in the form and function of internal buildings over time (Figure 1).

### Data sources

We have combined building vector data with land cover data to classify LCZ types in Beijing and combined them with OpenStreetMap data to obtain the Fifth Ring Road area. In addition, we downloaded diurnal ECOSTRESS LST data for different seasons and used ECOSTRESS CLOUD data for data filtering. At the same time, we selected images with better quality based on time requirements (In this article, we describe December, January, and February as winter; March, April, and May as spring; June, July, and August as summer; and September, October, and November as autumn). The data sources and times are shown in Table 1. To quantify the diurnal and seasonal thermal response of the LCZ, we superimposed the obtained LST data on the LCZ and produced corresponding graphs to highlight the differences between and within classes. We performed *t*-tests for the four seasons and diurnal LST, and a one-way ANOVA for the differences between LCZs.

**Table 1:** Data sources and descriptions.

**Data type**	**Descriptions**	**Time**	**Sources**
Building data	Buildings outlines, height and floor	2018	Baidu map
ECOSTRESS LSTE	Land surface temperature and emissivity data (70 × 70 m)	2019–2020	https://lpdaacsvc.cr.usgs.gov/appeears/
ECOSTRESS CLOUD	cloud mask product (70 × 70 m)	2019–2020	https://lpdaacsvc.cr.usgs.gov/appeears/
OpenStreetMap	Road location information	2021	https://www.openstreetmap.org/
Land cover data		2017	http://data.ess.tsinghua.edu.cn/

#### ECOSTRESS LST&E data

The ECOSTRESS experimental mission, which focuses on measuring plant temperature to better understand plant water requirements and how they relieve stress, consists of four levels of data, from which the level 2 product LST&E data and cloud mask product have been widely used for agricultural crop monitoring, volcanic surface temperature estimation, and studies of urban thermal environments with good accuracy (60, 61). In a systematic and comprehensive evaluation study of different satellite LST products, Li et al. (48) found that ECOSTRESS data had the highest consistency with ground-based observations during the day, with absolute deviations of <1.89°C at night. In this study, LST and cloud mask data for the study area were downloaded from the AppEEARS website (https://appeears.earthdatacloud.nasa.gov/explore), which allows the extraction of an individual dataset, as well as data reprojection (60, 62, 63). We selected diurnal LST images for each of the four seasons. After screening, eight high-quality images were selected for each day and night.

#### LCZ division

To maintain spatial resolution consistency with the LST data, the entire study area was divided into a 70 m × 70 m grid, and then divided into LCZs 1–9 based on building height and density. Based on the 2004–2020 urban master plan, most heavy industries were removed from the study area by 2016; therefore, LCZ10 is not divided (64). In addition, natural-type LCZ was classified based on land cover data. Due to the resolution difference, we reclassified the 10 m resolution land use data to 70 m in ArcGIS 10.5, and the type with the largest area of the pixel was taken as the attribute of the pixel. In addition, due to the lack of detailed geometric data on trees, LCZA (dense trees) and LCZB (sparse trees) were used as a mixed type, while scrubs, cultivated and grassland, and bare land in the land cover data were classified as LCZC, LCZD, and LCZF, respectively; streets and other impermeable surfaces were classified as LCZE, and water bodies were classified as LCZG (Table 2).

**Table 2:** LCZ types and description.

**LCZ type**	**Description**	**Examples**	**LCZ type**	**Description**	**Examples**
LCZ1	Compact high-rise (more than 9 floors)	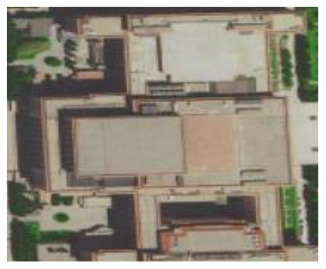	LCZA\B (Dense trees)	Dense\ Sparse coniferous forest and evergreen forest.	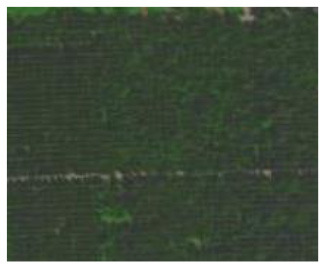
LCZ2	Compact mid-rise(4–8 floors)	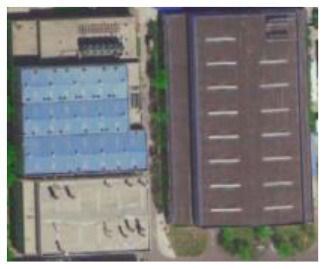	LCZC (Shrub)	Open arrangement of bushes, shrubs, and short, woody trees.	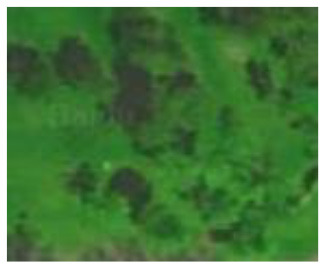
LCZ3	Compact low-rise(1–3 floors)	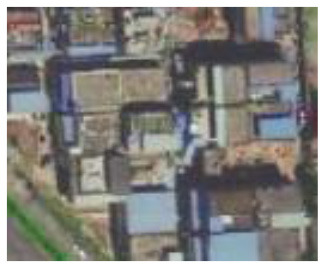	LCZD (Low plants)	Grassland or herbaceous plants crops. Few or no trees.	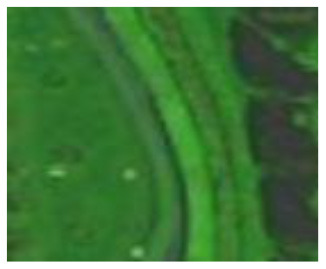
LCZ4	Open high-rise (more than 9 floors)	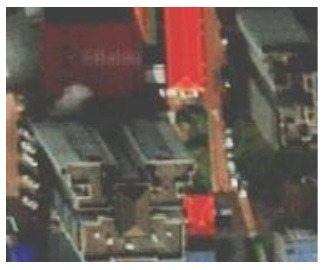	LCZE (Bare soil or paved)	Rock or impervious road surface virtually vegetation free	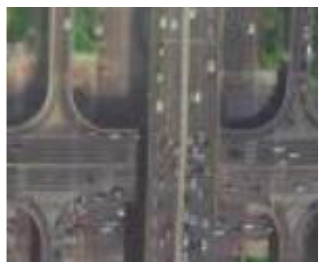
LCZ5	Open mid-rise (4–8 floors)	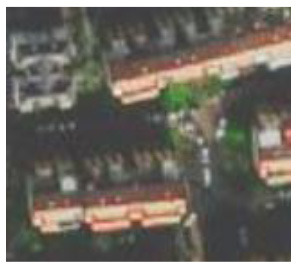	LCZF (Bare soil or sand)	Featureless landscape of soil or sand cover.	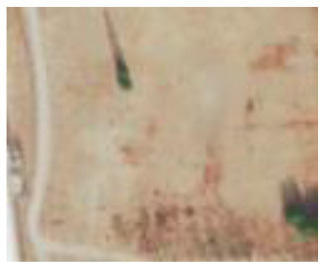
LCZ6	Open low-rise (1–3 floors)	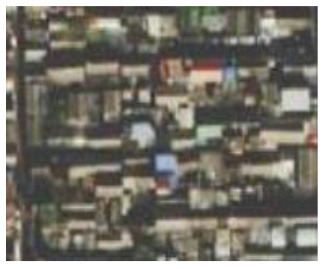	LCZG (Water)	Large, open water bodies or small bodies.	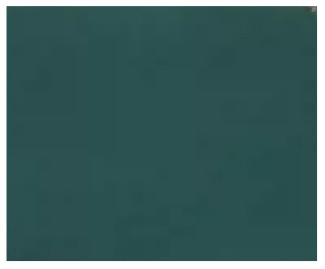
LCZ7	Sparse high-rise (more than 9 floors)	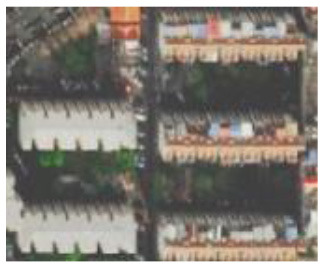	LCZ8	Sparse mid-rise (4– 8 floors)	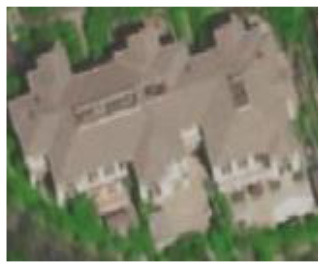
LCZ9	Sparse low-rise (1–3 floors)	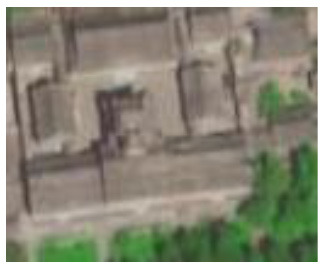	LCZ1–3: building density >0.4. LCZ4–6: building density is between 0.2 and 0.4. LCZ7–9: building density <0.2.		

## Results

### LCZ classification results

**Figure 2:**
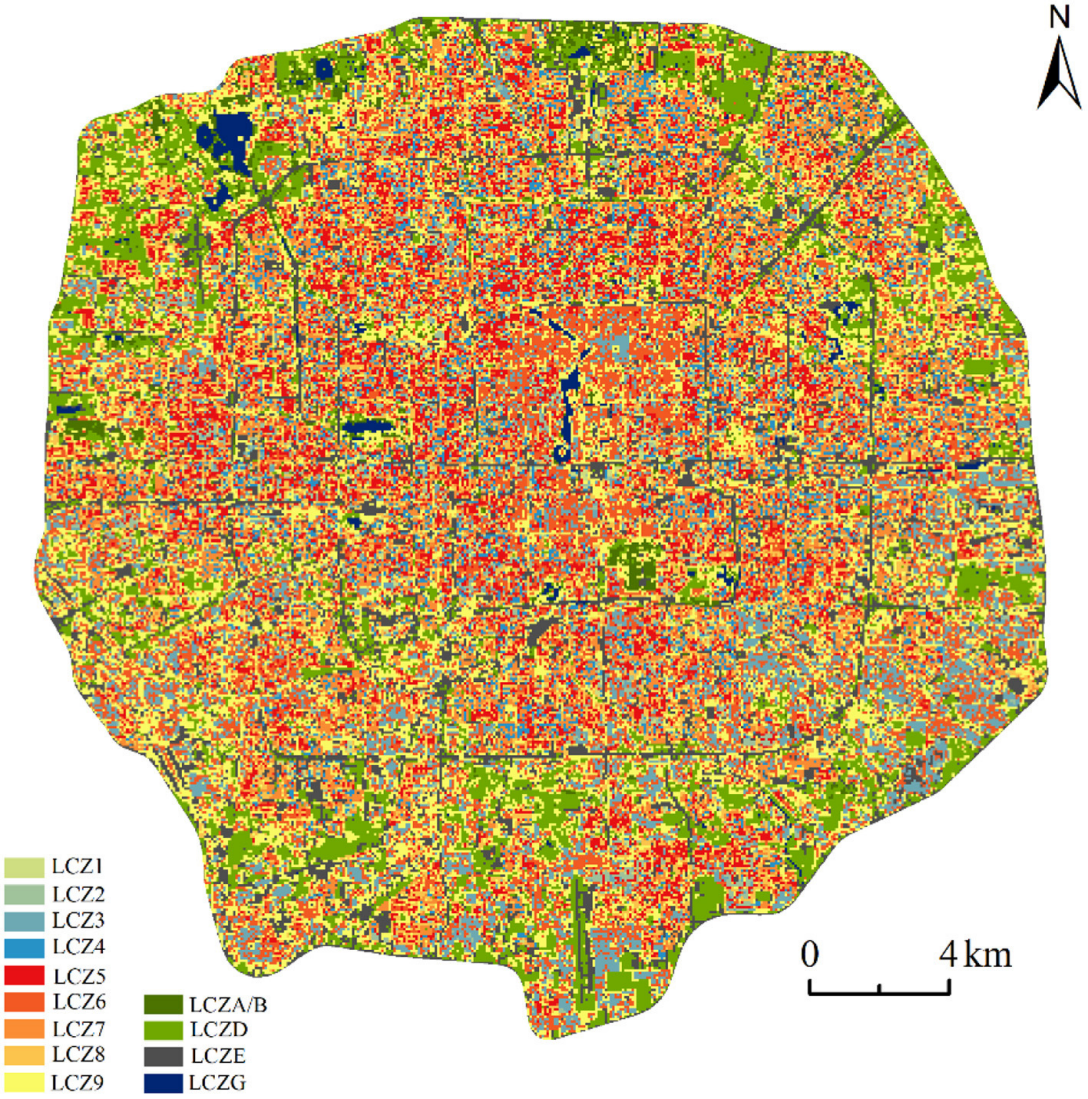
Urban local climate zone (LCZ) map of the study area.

**Figure 3:**
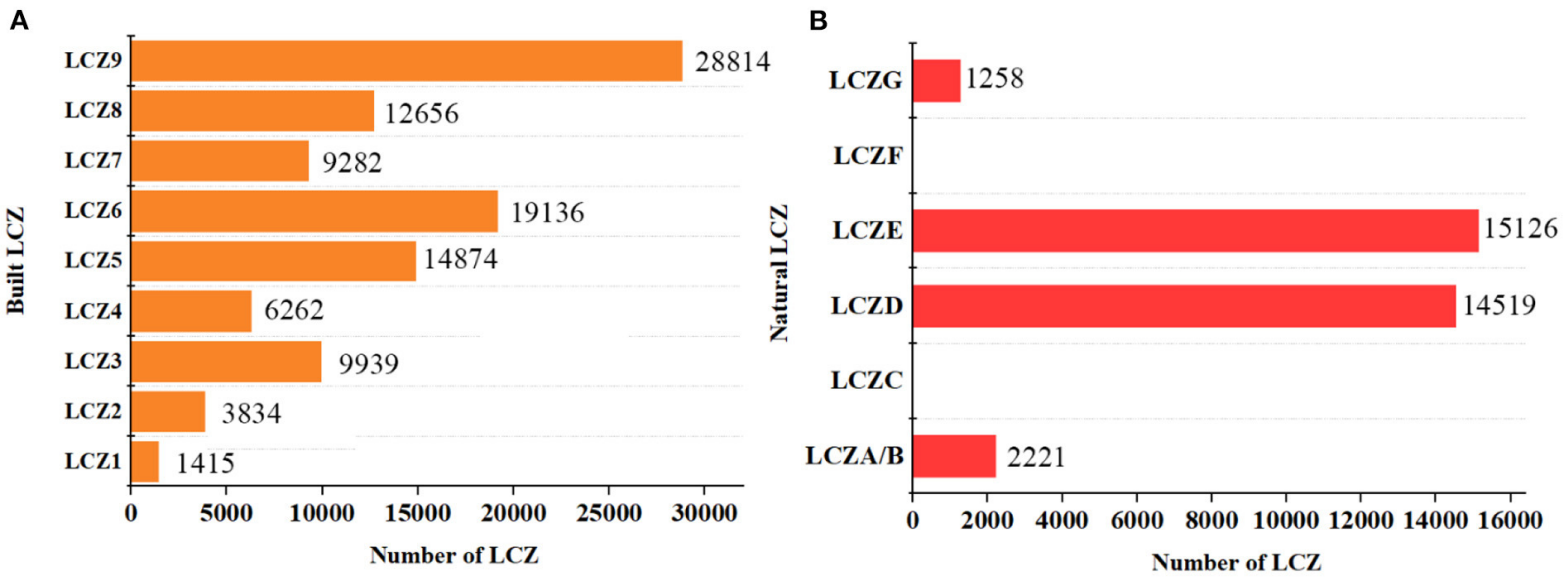
Number of built and natural type LCZs in the study area, **(A)** is the number of buildings type LCZs; **(B)** is the number of nature type LCZs.

Figure 2 shows the LCZ classification results of the study area, which is divided into 13 LCZ types. Overall, the area within the Fifth Ring Road of Beijing was mostly covered by building-type LCZs, while natural-type LCZs were smaller in size. It also shows a decreasing trend from the center to the periphery, which is attributed to the height limit policy of Beijing. Meanwhile, combining with Figure 3, it can be found that LCZ9(sparse low-rise building) and LCZ1(compact high-rise building) are the dominant and the lowest among building types, with 28,814 and 1,415 pixels, respectively, while only four natural types are obtained, which are LCZA/B, LCZD, LCZE and LCZG. LCZC and LCZF did not appear in the entire study area. In addition, the combination of Google Earth satellite images shows that LCZA/B and LCZG are spatially distributed in line with the location of the park while LCZE mainly consists of a network of ring roads.

### Diurnal and seasonal LST variation

**Figure 4:**
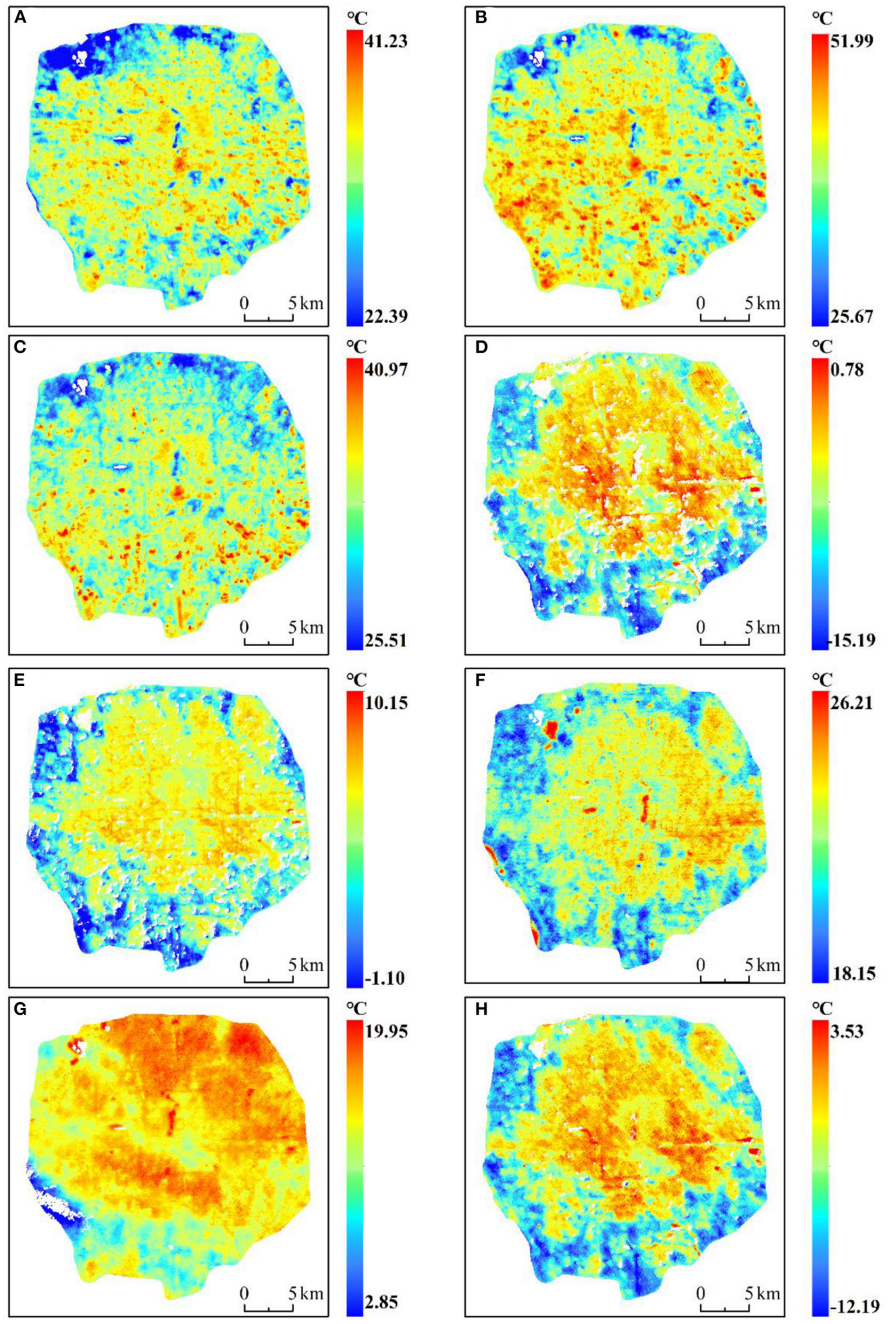
Diurnal and seasonal land surface temperature (LST) variation in the 5th Ring Area, Beijing, China. **(A–D)** Daytime LSTs during spring, summer, autumn, and winter, respectively. **(E–H)** Night-time LSTs during spring, summer, autumn, and winter. All times are in Beijing time (UTC+8). The white areas in the map indicate missing data **(G)** and areas with clouds removed.

Figure 4 shows the spatial distribution of day and night LST in the study area during four seasons. Figures 4A–D show daytime LST, and Figures 4E–G show nighttime LST during four seasons. The spatial distribution of land surface temperature is different between seasons and day and night. Therefore, we explored the diurnal variation of urban thermal environment. The daily LSTs of four seasons were measured and *t*-test was performed, and the results showed that LSTs was significant (*P* < 0.05). The results of LST in daytime were in the order of summer (40.44°C) > spring (34.72°C) > autumn (31.83°C) > winter (−9.45°C), and the highest LST at night was in summer (21.69°C). followed by autumn (14.87°C), spring (5.37°C) and winter (−6.49°C) (Table 3). In addition, combined with Figure 2, it can be discerned that the high temperature areas during the daytime are mainly concentrated in the building area, because the building materials have high heat absorption, leading to higher surface temperature. However, the areas with low temperature are concentrated in natural landscapes with trees and water bodies. Combining Figure 1C and Google Earth satellite images, it can be discerned that these areas are mainly composed of parks, such as Beihai Park and Shichahai Park in the center, the Old Summer Palace and the Summer Palace in the northwest, and the Olympic Park in the north. The results show that urban park areas usually have lower LST, which can mitigate the UHI effect to some extent, which is consistent with previous studies (41). In addition, different landscapes in urban parks also have different effects on human comfort (65). The nighttime temperature in the study area was lower than the daytime temperature during all the seasons (Table 3). In addition, the water body absorbs a large amount of heat during the day, while at night, due to its high specific heat capacity, it is lost more slowly, resulting in higher temperatures than surrounding buildings, especially during summer (Figure 4F).

**Table 3:** Statistical results of diurnal surface temperature in different seasons.

**Time**	**Max** **(°C)**	**Min** **(°C)**	**Avg** **(°C)**
Spring daytime	38.89	30.95	34.72
Summer daytime	46.58	34.55	40.44
Autumn daytime	35.73	27.98	31.83
Winter daytime	−4.47	−14.51	−9.45
Spring nighttime	9.51	1.42	5.37
Summer nighttime	24.35	19.07	21.69
Autumn nighttime	18.83	11.20	14.87
Winter nighttime	−1.85	−11.09	−6.49

### Diurnal and seasonal LST distribution in LCZs

**Figure 5:**
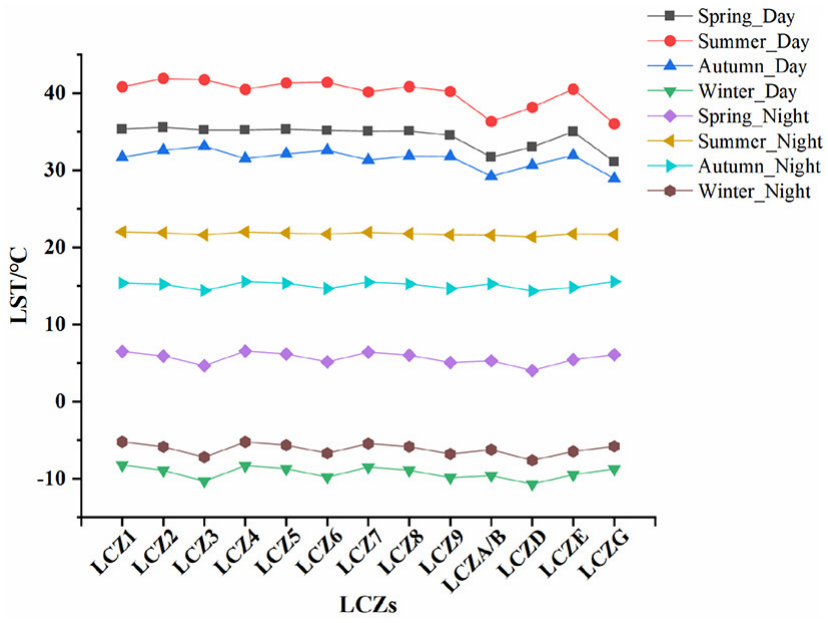
Temperature variation of each day and night LCZ during the different seasons.

**Figure 6:**
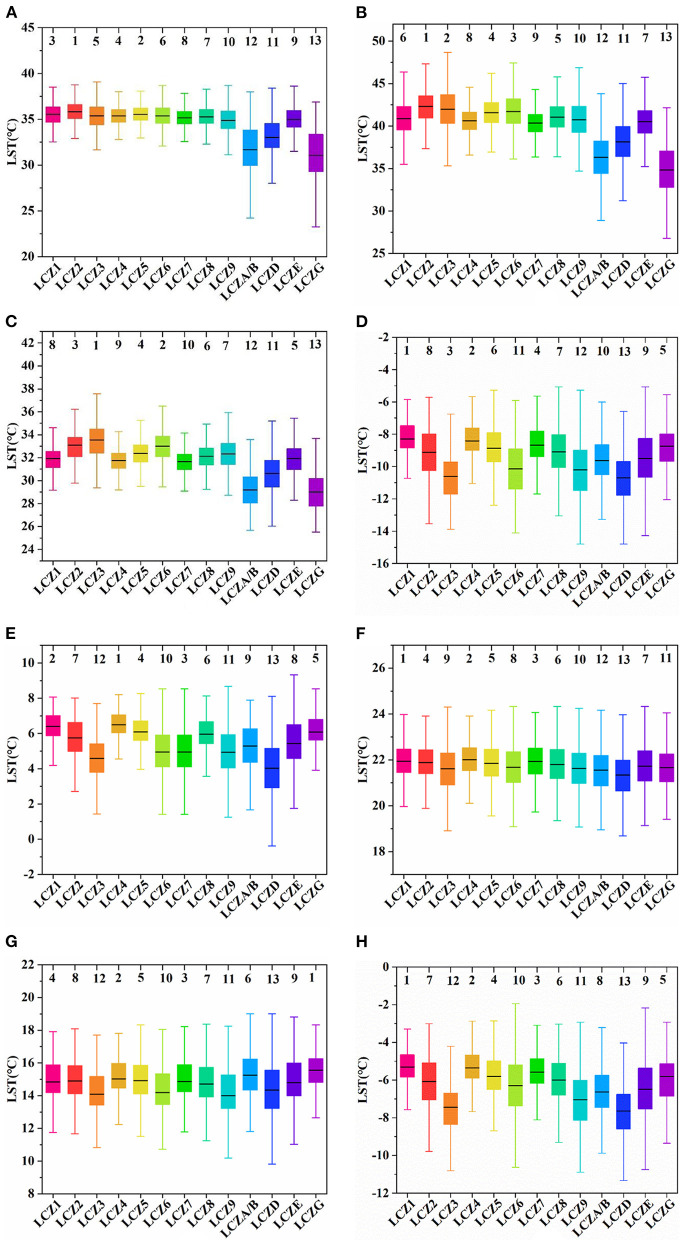
**(A–D)** Box line plots for each daytime LCZ during spring, summer, autumn, and winter, respectively; **(E–H)** Box line plots for each night-time LCZ during spring, summer, autumn, and winter, respectively. The lines in the box represent the average, and the numbers above represent LCZs ranked according to average LST (the warmest to the coldest zones from 1 to 13).

The statistical analysis results show that the LST and LCZ spatial distributions were highly consistent. We found that daytime LST was higher than night-time LST for each LCZ during the different seasons (Figure 5), and the average summer LST for each LCZ was higher than that of the other seasons, except for winter. Furthermore, daytime LST for each LCZ was lower than night-time LST during winter season, indicating that it was colder during winter. In addition, we further analyzed the diurnal LSTs of each LCZ during the different seasons and visualized their distribution through box line plots. Figures 6A–D show the box line plots for daytime LCZs during spring, summer, autumn, and winter, respectively, while Figures 6E–H show the box line plots for their respective night-time LCZs. The results were confirmed using one-way ANOVA (daytime values: *F* = 3259.696, *P* < 0.01; night-time values: *F* = 3514.9, *P* < 0.01). However, since LSTs obtained were for different seasons, we ranked each LCZ according to its average temperature. In daytime (Figures 6A–D), the highest LSTs during spring, summer, autumn, and winter were 35.54°C (LCZ2), 41.90°C (LCZ2), 33.09°C (LCZ3), and −8.24°C (LCZ1), respectively. Thus, the LSTs of building-type LCZs were generally higher than those of natural-type LCZs. The LST of the natural LCZG (water body) was the lowest in all seasons (except winter). At night, the LSTs of building-type LCZs did not show an obvious consistent pattern of change, while the LSTs of the natural LCZD (grassland) were the lowest during spring, summer, autumn, and winter (4.02°C, 21.33°C, 14.34°C, and −7.64°C) respectively.

**Figure 7:**
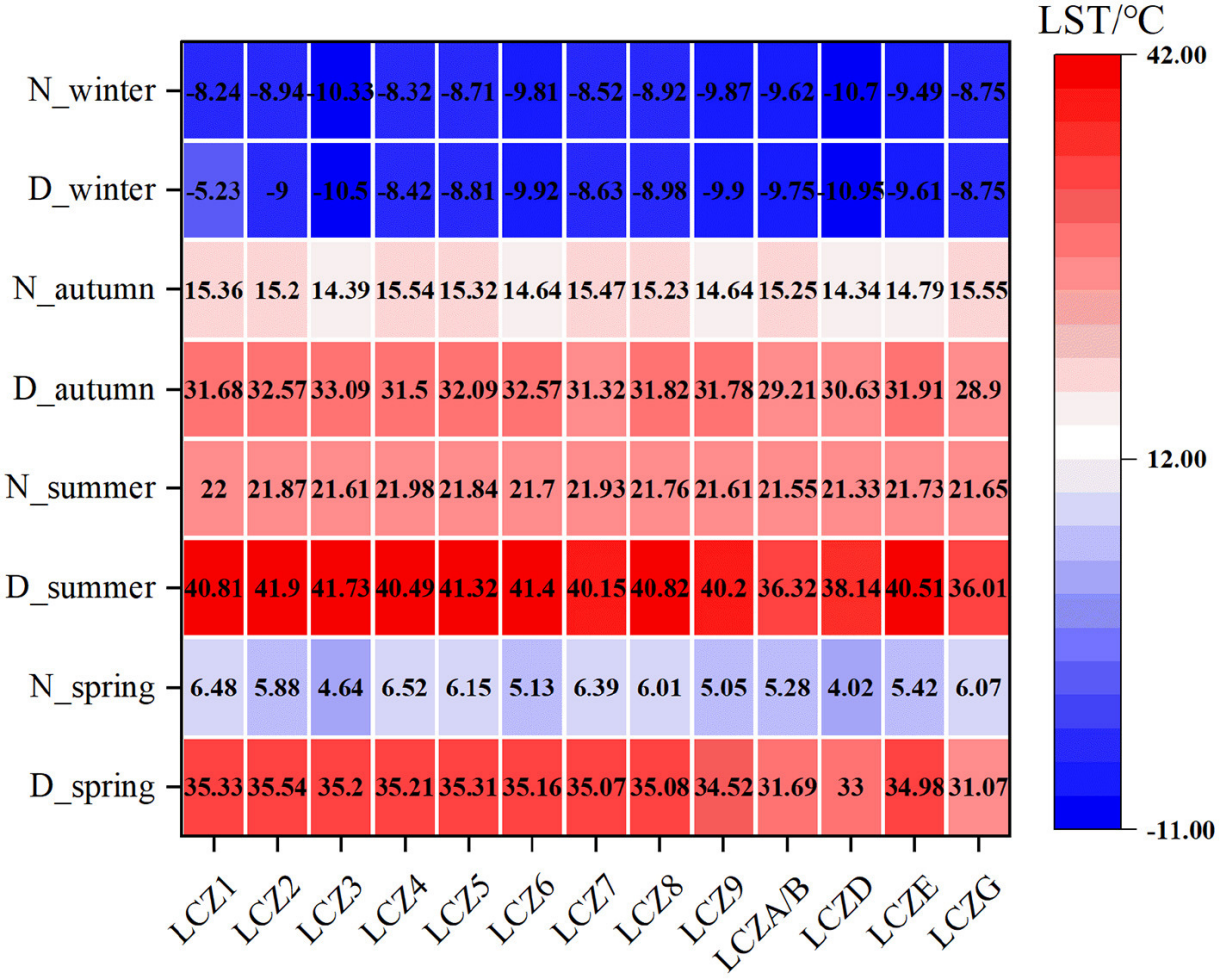
Diurnal variation of the same LCZ in different seasons.

Meanwhile, we studied the distribution of day and night surface temperature of the same LCZ in different seasons. The results are shown in the Figure 7. It can be seen from the figure that the daytime average surface temperature of all LCZ was the highest in summer, with LCZ3 reaching 41.73°C, and the lowest LCZG reaching 36.01°C, which indicates that it is necessary to formulate corresponding heat mitigation measures in summer. For all LCZ, the surface temperature in daytime was higher than that at night in the same season, which was mainly due to the large amount of heat absorbed by all LCZ during daytime and heat loss at night. In addition, in the same season, the LCZ surface temperature had significant difference between day and night.

## Discussion

### Changes in LST as a function of LCZ

We inventoried the seasonal variations in the urban thermal environment as well as diurnal variations due to intra-city heterogeneity using the LCZ framework in the Fifth Ring Road area of Beijing. Overall, our results showed significant differences in the LSTs of the LCZs between the daytime and night-time as well as in different seasons. The temperature differences between LCZs (LCZ2 and LCZG) with large differences in surface material and cover type reached 5.89°C during the daytime in summer, while the differences were generally <2.50°C at night (except in winter). Compared with the building type LCZ, the natural type LCZ had a lower surface temperature (except in winter). Taking summer as an example, the daytime surface temperature was LCZG(13) < LCZA/B(12) < LCZD(11) < LCZE(7), the nighttime surface temperature presented LCZD(13) < LCZA/B(12) < LCZG(11) < LCZE(7), which indicates that in the hot summer, the vegetation and water bodies have lower surface temperatures in both daytime and night-time, related studies have shown that vegetation and water bodies are important components of urban heat island mitigation in cities (66). Zhou et al. (34) quantified diurnal and seasonal UHII of 32 major Chinese cities to analyze their spatial variability and influencing factors, and suggested that a variety of strategies are needed to effectively mitigate the UHI effect. However, as cities grow and become increasingly heterogeneous, targeted solutions are required. LCZ solutions can be classified into different types based on surface materials, ground cover, and the influence of human activities. A study by Zhang et al. (67) on the temporal and spatial variability of UHI and urbanization in Beijing showed that LCZ conversion had a significant impact on surface UHII. In addition, Quan et al. (68) conducted an LCZ classification of Beijing, combining MODIS data with wind and cloud data at 100 m; the results showed significant differences in the LCZ diurnal thermal response with seasonal changes. It is therefore important to use higher resolution data to study diurnal differences and seasonal variations in urban thermal environments from the perspective of the LCZ. In addition, as a core area of Beijing, the Fifth Ring Road area was divided into LCZ by pixel-based method, and the whole study area was divided into 13 types. Compared with the method of LCZ division based on community units, this pixel-based method could show the LST differences of each LCZ more clearly. Further understanding of the spatial heterogeneity of urban LST caused by the complex intra-city landscape is required to provide certain references to improve local climate and for reasonable planning practice.

### Use of ECOSTRESS data

The change of urban environment has an important impact on the improvement of human settlements. Therefore, we used LCZ scheme combined with ECOSTRESS LST data to study the urban thermal environment and analyze its changes during different seasons and between day and night. ECOSTRESS data can obtain the surface temperature of most parts of the world from different time periods with the advantages of multi time points and high resolution. It makes up for the deficiency of traditional ASTER and MODIS data timing observations. Taking the Fifth Ring Road in Beijing as an example, we show the unique advantages of ECOSTRESS LST data in analyzing the urban thermal environment, and provide a new perspective for the study of urban thermal environment. When there are data from multiple time periods from the same area, we can study the diurnal variation of surface temperature in urban areas more accurately; such as the diurnal variation in surface temperature and surface UHII in three cities in India was analyzed by Siddiqui et al. (69) using MODIS LST data. Lu et al. (70) combined MODIS and Landsat images to analyze diurnal differences and seasonal characteristics of surface UHII variability in Hefei, China. Compared with MODIS LST data, ECOSTRESS LST data show higher temporal and spatial resolution and can more accurately reflect temperature differences. Our study shows that LST spatial distribution in urban areas varied between day and night, which is consistent with the findings of previous studies (71, 72). The daytime differences in LST are mainly related to the type of ground cover and material; for instance, building materials and impermeable surfaces tend to absorb more energy than vegetation. The difference in nocturnal LST is mainly caused by the discharge of accumulated energy during the day (73); water bodies that absorb large amounts of heat during the day release it at night, leading to higher temperatures at night than during the day.

### Limitations

This study has some limitations. We used the ECOSTRESS LST data and LCZ framework to study the seasonal and diurnal differences in LST in Beijing. First, when LCZ segmented building vector data, only existing data were used for segmentation because the latest building data was not available at that time. There may be some differences with existing building forms. In addition, although ECOSTRESS can perform repeated observations of the same area within 3–5 days, due to the impact on data quality, the day and night LST data are not guaranteed to belong to the same day, so there may be some uncertainty in the data accuracy. At the same time, in order to avoid the influence of extreme weather and diurnal variation of LCZs, high-quality and time-consistent images should be used in future studies.

## Conclusions

We analyzed the thermal characteristics of different LCZs in the Fifth Ring Road area of Beijing on both diurnal and seasonal time scales, constructed LCZs for the study area using GIS methods, and downloaded diurnal ECOSTRESS LST data for all seasons to analyze the diurnal and seasonal LST variations within and between the LCZ classes. The main conclusions can be summarized in three points.

(1) Overall, the Fifth Ring Road area of Beijing is mainly composed of building-type LCZs, while natural-type LCZs are scarce. LCZ9 (sparse low-rise buildings) is an important part of the built-up LCZs. In addition, natural LCZs, such as LCZA/B (woodland), LCZD (grassland), and LCZG (water body), mainly comprise park landscapes, as determined in combination with Google Earth satellite images.
(2) There were significant differences in LSTs between the seasons and between day and night; specifically, daytime LST showed a summer > spring > autumn > winter trend, while the night-time trend was summer > autumn > spring > winter. All LST results were highest during summer and lowest during winter. In addition, high-temperature areas during the day were concentrated in building areas, while low-temperature areas were concentrated in natural landscape areas comprising trees and water bodies, most of which included park landscapes. At night, the temperature in the study area was lower than the daytime temperature in all the seasons.
(3) The spatial distribution of LST and LCZ was highly consistent. In addition, the average temperature of the LCZs was ranked according to the seasonal and diurnal LSTs, and the highest LSTs found during the four seasons were in LCZ2 (spring), LCZ2 (summer), LCZ3 (autumn), and LCZ1 (winter). The LSTs of building-type LCZs were higher than those of natural-type LCZs, while the LST of LCZG (water body) was lowest in all seasons except winter. At night, there was no obvious pattern of LST change in building-type LCZs; in contrast, LCZD ranked last among the natural-type LCZs in all seasons, indicating that its average LST was the lowest. Therefore, natural landscapes, such as woodland, grassland, and water bodies, should be reasonably laid out in urban planning to improve the urban environment and quality of life.

Based on the LCZ framework, this paper analyzes the diurnal surface temperature changes in the Fifth Ring Road area of Beijing, and has a more comprehensive understanding of the surface temperature changes under different ground covers during different seasons and day and night. With the continuous development of the night economy, while studying the changes of the urban thermal environment during the day, the changes in the urban thermal environment at night should also be paid more attention. The ECOSTRESS LST product, It can observe the change of night surface temperature with higher resolution, and use the advantage of high time resolution to obtain the surface temperature at multiple time points in a certain area. In future research, the understanding of diurnal surface temperature changes in urban areas can be further improved. Extensive ECOSTRESS data and more detailed LCZ classification will deepen the understanding of diurnal changes in urban thermal environment in the future, and shall provide references for future urban planning and the formulation of heat mitigation strategies.

## Data availability statement

The original contributions presented in the study are included in the article/supplementary material, further inquiries can be directed to the corresponding author/s.

## Author contributions

JY contributed to all aspects of this work. ZS wrote the main manuscript text. LW, FL, GW, XX, and JX conducted the experiment and analyzed the data. All authors reviewed the manuscript. All authors contributed to the article and approved the submitted version.

## Funding

This research study was supported by the National Natural Science Foundation of China (grant no. 41771178 and 42030409), the Fundamental Research Funds for the Central Universities (grant no. N2111003), Basic Scientific Research Project (Key Project) of the Education Department of Liaoning Province (grant no. LJKZ0964), the Second Tibetan Plateau Scientific Expedition and Research Program (STEP) (grant no. 2019QZKK1004), and Innovative Talents Support Program of Liaoning Province (grant no. LR2017017).

## Conflict of interest

The authors declare that the research was conducted in the absence of any commercial or financial relationships that could be construed as a potential conflict of interest.

## Publisher's note

All claims expressed in this article are solely those of the authors and do not necessarily represent those of their affiliated organizations, or those of the publisher, the editors and the reviewers. Any product that may be evaluated in this article, or claim that may be made by its manufacturer, is not guaranteed or endorsed by the publisher.
